# Mir-141-3p Regulates Apoptosis and Mitochondrial Membrane Potential via Targeting Sirtuin1 in a 1-Methyl-4-Phenylpyridinium in vitro Model of Parkinson's Disease

**DOI:** 10.1155/2020/7239895

**Published:** 2020-11-06

**Authors:** Yumin Zheng, Li Dong, Na Liu, Xiaoguang Luo, Zhiyi He

**Affiliations:** ^1^Department of Neurology, The First Affiliated Hospital of China Medical University, Shenyang, China; ^2^Department of Neurology, The Fourth Affiliated Hospital of China Medical University, Shenyang, China; ^3^Department of Neurology, Nanfang Hospital, Southern Medical University, The Second Affiliated Hospital of Jinan University, Guangzhou, China; ^4^Shenzhen People's hospital, Shenzhen, China

## Abstract

**Objectives:**

Parkinson's disease (PD) is a common neurodegenerative disease characterized by the loss of midbrain dopaminergic neurons in the substantia nigra. The present study investigated miR-141-3p/sirtuin1 (SIRT1) activity in a 1-methyl-4-phenylpyridinium- (MPP+-) induced PC12-cell model of PD.

**Methods:**

PC12 cells were exposed to MMP+ following induction of differentiation by nerve growth factor (NGF). miR-141-3p and SIRT1 expressions were examined using RT-qPCR and western blot. Cell viability was evaluated using the MTT assay. Apoptosis percentage, reactive oxygen species (ROS) production, and mitochondrial membrane potential (*Δψ*m) were evaluated using flow cytometry. Expression of Nuclear factor-kappa B- (NF-*κ*B-) related proteins was determined by western blot. Bioinformatic analysis, RT-qPCR, and luciferase reporter assay were used to confirm the interaction between miR-141-3p and SIRT1.

**Results:**

miR-141-3p was upregulated, and SIRT1 was downregulated in MPP+-treated PC12 cells. MPP+ treatment also upregulated nitric oxide synthase 1 (Nos1) and *α*-synuclein. miR-141-3p induced apoptosis, oxidative stress, mitochondrial dysfunction, and downregulated the SIRT1 mRNA expression. The luciferase reporter assay showed that SIRT1 was the target of miR-141-3p. SIRT1 transfection attenuated apoptosis, ROS production and maintained *Δψ*m. SIRT1 also downregulated Nos1, tumor necrosis factor-*α* (TNF-*α*), interleukin 1 beta (IL-1*β*), interleukin 6(IL-6) and upregulated B cell lymphoma 2 (Bcl-2) protein. In addition, SIRT1 activator resveratrol blocked the effects of miR-141-3p mimic on Nos1, *α*-synuclein, and mitochondrial membrane potential. SIRT1 inhibitor sirtinol reversed the biological effects of miR-141-3p.

**Conclusion:**

Increased miR-141-3p induced apoptosis, oxidative stress, and mitochondrial dysfunction in MPP+-treated PC12 cells by directly targeting the SIRT1 expression. Our study provided a potential therapeutic strategy for PD.

## 1. Introduction

Parkinson's disease (PD) is one of the most common neurodegenerative diseases worldwide [1–3]. The pathological feature of Parkinson is loss of midbrain dopaminergic neurons in the substantia nigra. To date, the pathophysiologic mechanisms underlying the loss of dopaminergic neurons remain elusive. Although various hypotheses (including mitochondrial dysfunction [4, 5], oxidative stress [6], and inflammation [7, 8]) have been proposed and tremendous progress has been made to improve life quality, the therapeutic effects are not satisfactory.

The sirtuin family proteins are histone deacetylases that play important roles in the longevity effects caused by energy limitations. The SIRT1 gene is located on chromosome 10q21.3 and is widely expressed in human tissues including the brain. SIRT1 targets nonhistone and histone proteins involved in the regulation of physiological processes including DNA repair, chromosome stability, mitochondrial function, antioxidant defense, and apoptosis [9, 10]. SIRT1 could inhibit toxin-induced cell apoptosis through deacetylating numerous core transcription factors in PD [11]. SIRT1 has been shown to regulate the mitochondrial function via PPARG coactivator-1 alpha (PGC-1*α*) ,and to reduce oxidative stress in response to forkhead box protein O (FOXO) family members [12, 13].

microRNAs (miRNAs) are small noncoding RNAs consisting of approximately 17-25 nucleotides that regulate the expression of target genes through binding to the 3' untranslated regions (3′-UTRs). An increasing number of studies in PD models and brain tissue of PD patients have associated the dysregulation of miRNAs with the pathophysiological process of PD. A recent study reported that miR-410 was decreased in SH-SY5Y and PC12 cells induced by 6-hydroxydopamine and that miR-410 overexpression alleviated injury induced by 6-hydroxydopamine, restored cell viability, and inhibited apoptosis and ROS production [14]. Another report found upregulation of multiple miRNAs, including miR-34a-5p, miR-9-5p, and miR-141-3p, in the MPP+-induced PC12 PD model [15]. To date, the biological function and mechanisms of miR-141-3p in the PD development have not been studied.

In this study, we aimed to explore the roles and molecular mechanisms of the miR-141-3p/SIRT1 axis in the regulation of apoptosis, oxidative stress, and mitochondrial function by using the MPP+-intoxicated PC12 cell model.

## 2. Materials and Methods

### 2.1. Cell Culture and Treatment

PC12 cells were purchased from the American Type Culture Collection (ATCC, Manassas, VA, USA) and were cultured in Dulbecco's Modified Eagle Medium (DMEM, Gibco, Carlsbad, CA, USA) containing 10% heat inactivated horse serum, 5% fetal bovine serum (FBS, Gibco, Carlsbad, CA, USA), and 100 U/ml penicillin-streptomycin (Gibco, Carlsbad, CA, USA) at 37°C, 5% CO2, and saturated humidity. PC12 cells were differentiated in the DMEM containing 1% heat inactivated horse serum and 100 U/ml penicillin-streptomycin. Cells were treated with recombinant human NGF (R&D, USA) at the concentration of 50 ng/ml for 7 days. ThePD model was induced by incubation of differentiated PC12 cells with 800 *μ*M of MPP+ (Sigma-Aldrich, MO, USA) for 24 hours.

### 2.2. 3-(4,5-dimethylthiazol-2-yl)-2,5-diphenyltetrazolium (MTT) assay

The viability of PC12 cells was determined by MTT. Cells were incubated with MTT (Sigma-Aldrich, MO, USA) for 4 hours. DMSO (Sigma-Aldrich, MO, USA) was added into each culture well to dissolve the precipitate after the supernatant was discarded and the cell viability index was determined by measuring the optical density at 490 nm using a microplate reader (Infinite F50, TECAN, Männedorf, Switzerland). For calculation of relative viability, viability of MPP+-treated differentiated PC12 cells was divided by the viability of differentiated PC12 cells without MPP+ treatment.

### 2.3. RT-qPCR

Total RNA was extracted from PC12 cells using TRizol (Life technology, MO, USA) and quantified with a nanodrop spectrometer (Thermo Fisher, CA, USA). cDNA synthesis for SIRT1/tyrosine hydroxylase (TH) and miR-141-3p was performed using PrimeScript RT Master Mix (Takara, Dalian, China) and PrimeScript RT Master Kit (Takara, Dalian, China), respectively, according to the manufacturer's instructions. Gene amplification was performed using SYBR Green Master Mix (Applied Biosystems, MA, USA) on an ABI 7500 instrument (Applied Biosystems, MA, USA). The miR-141-3p expression was normalized by U6, and the SIRT1/TH expression was normalized by GAPDH. Calculation of miR-141-3p, SIRT1, and TH was performed according to the 2^-△△ct^ method. All reactions were performed in triplicate. Oligo 7 primer analysis software was used for primer design, and the sequence of primers was listed as follows: SIRT1 for 5′-for AAGGAGCAGATTAGTAAGC-3′, SIRT1 rev 5′-TAGAGGATAAGGCGTCAT-3′; TH for 5′-CAGCAGCAGCAGCGGTAG-3′, TH rev 5′-CAGGGAGAAGAGCAGGTTGAG-3′; GAPDH for 5′-GCTGGTCATCAACGGGAAA-3′, GAPDH rev 5′-CGCCAGTAGACTCCACGACAT-3′; miR-141-3p for 5′-CGGGCTAACACTGTCTGGTAAAG-3′, miR-141-3p rev 5′-GTGCAGGGTCCGAGGTATTC-3′; U6 for 5′-CTCGCTTCGGCAGCACA-3′, U6 rev 5′-AACGCTTCACGAATTTGCGT-3′.

### 2.4. Cell Transfection

PC12 cells were plated in 6-well plates and cultured at 37°C till 80% confluence before transfection. SIRT1 plasmid and empty plasmid were purchased from Origene (Rockville, MD, USA), and they were transfected into PC12 cells using the Lipofectamine 3000 Transfection Reagent (Invitrogen, Carlsbad, CA, USA) according to the manufacturer's instruction. SIRT1 siRNA and nontargeting siRNA were purchased from Dharmacon (Horizon, Cambridge, UK). The miR-141-3p mimic, mimic control, miR-141-3p inhibitor, and inhibitor control were purchased from Ribobio (Guangzhou, China). The small RNA sequences were transfected into PC12 cells using the Dharmafect1 Transfection Reagent (Horizon, Cambridge, UK) according to the manufacturer's instructions. Briefly, 1 *μ*g plasmid, 3 *μ*l lipofectamine 3000, and 100 *μ*l serum-free medium were incubated at room temperature for 15 minutes prior to transfection. The Dharmafect1 Transfection Reagent (5 *μ*l) combined with RNA sequences at a concentration of 100 nM were incubated at room temperature for 20 minutes. The mixture was then added into the PC12 cultures and incubated for 6 hours before replacng the medium with fresh medium with PBS for the future culture.

### 2.5. Western Blot

Cell lysate was prepared by scraping PC12 cells in pierce luciferase cell lysis buffer (Invitrogen, Carlsbad, CA, USA). Protein concentration was determined by the BCA method. Protein samples (50 *μ*g) were separated on a 10% SDS-PAGE gel and transferred to the PVDF membranes (Millipore, Boston, MA, USA). The membranes were incubated with BSA (Biofroxx, Guangzhou, China) at room temperature for 90 minutes to block nonspecific binding incubation with corresponding primary antibodies against SIRT1 (1 : 1000, Abcam, Cambridge, UK), *α*-synuclein (1 : 1000, Cell Signaling Technology, MA, USA), Nos1 (1 : 800, Abcam, Cambridge, UK), TNF-*α* (1 : 1000, Abcam, Cambridge, UK), p-I*κ*B (1 : 1000, Cell Signaling Technology, MA, USA), and *β*-actin (1 : 2000, Cell Signaling Technology, MA, USA) at 4°C overnight. The membranes were then incubated with horseradish peroxidase-conjugated secondary antibodies for 2 hours. The membranes were visualized by ECL Chemiluminescent Substrate Reagent Kit (Thermo Fisher, CA, USA) and DNR Bio-Imaging System (Jerusalem Israel). Image J software was used for blot densitometry.

### 2.6. Apoptosis Assay

Cell apoptosis was investigated by dual staining with annexin V-FITC and propidium iodide (BD Pharmingen, NJ, USA). PC12 cells were plated at a density of 1 × 10^5^ per well in 6-well plates and cultured at 37°C for 48 hours. Trypsin was used to dissociate cells from the dishes in which differentiated PC12 cells were being cultured. Cells were centrifuged at 1000 rpm for 10 minutes. Supernatant was removed, and the cells were washed twice with PBS buffer. The cells were suspended in 400 *μ*l binding buffer supplemented with 5 *μ*l annexin V. The mixture was incubated at room temperature in the dark for 10 minutes. PI solution (5 *μ*l) was added, and the mixture was incubated for 15 minutes in the dark at room temperature. Cell apoptosis percentage was determined using NovoCyte flow cytometry(ACEA, USA).

### 2.7. Immunofluorescence

Cells on Lab-Tek Chamber Slides were fixed with 4% paraformaldehyde in PBS, permeabilized using 0.1% Triton, and then incubated with primary antibody against *α*-synuclein and AlexaFluro 488-conjugated secondary antibodies (Molecular Probes, USA). Photos was taken using an Olympus BX53 microscope (Olympus, Japan).

### 2.8. ROS Production Assay

The CellROX Deep Red Reagent (Invitrogen, Carlsbad, CA, USA) was used to analyze ROS production in PC12 cells according to the manufacturer's protocols. Briefly, the cells were treated with trypsin, washed with PBS buffer, and resuspended with 5 *μ*M of Deep Red Reagent. The cells were incubated for 30 minutes, centrifuged at 1300 rpm for 10 minutes, and resuspended with 300 *μ*l PBS buffer. ROS production was analyzed by detecting fluorescent intensity using NovoCyte flow cytometry (ACEA, USA).

### 2.9. Mitochondrial Membrane Potential

*Δψ*m was analyzed via measuring the fluorescent intensity of cells stained with JC-1 dye (ab141387, Abcam, Cambridge, UK). The PC12 cells were treated with trypsin, washed with PBS buffer, and resuspended with 300 *μ*l DMEM medium. JC-1 dye (1 *μ*g/ml) was added, and the cells were incubated for 45 minutes. After resuspending the cells in PBS buffer, the fluorescence intensity of the mixture was analyzed by NovoCyte flow cytometry (ACEA, USA).

### 2.10. Luciferase Reporter Assays

Luciferase reporter plasmids containing the Sirt1 3′-UTR (WT and mutant) were constructed using the psiCHECK-2 dual luciferase vector (Promega, Madison, WI, USA).

Briefly, Sirt1 3′-UTR fragment containing predicted miR-141-3p target sites (5′-UUUGAAAUACAAAAACAGUGUUU-3′) was amplified by PCR. The primers are as follows: forward, CCGCTCGAGGGCATATGTTTTGTAGACCGTT (underlined letters indicate the *Xho*I site) and reverse, 5′-ATAAGAATGCGGCCGCTTTCCTGAGGCATTTAGTG-3′ (underlined letters indicate the *Not*I site). The *Xho*I and *Not*I-digested PCR product was then cloned into a psiCHECK-2 vector to generate the psiCHECK-2-Sirt1-3′-UTR-wild-type. The fragment with mutated predicted miR-141-3p target sites (5′-UUUGAAAUCCAAAAACCCCCUUU-3′) was directly synthesized (Sangon Biotech, Shanghai, China), digested with *Xho*I and *Not*I, and cloned into the psiCHECK-2 vector to generate the psiCHECK-2-Sirt1-3′-UTR-mutant. Then, 100 ng of the psiCHECK2-SIRT1 vector was cotransfected into PC12 cells with miR-141-3p mimic or negative control using the Lipofectamine 3000 Transfection Reagent (Invitrogen, Carlsbad, CA, USA), with 10 ng of Renilla luciferase reporters used as an internal control. The luciferase activities were assessed using a Dual-luciferase Reporter Assay Kit (Promega, WI, USA) according to the manufacturer's protocols.

### 2.11. Enzyme-Linked Immunosorbent Assay (ELISA)

The levels of proinflammatory cytokines TNF-*α*, IL-1*β*, and IL-6 in the culture medium were detected by ELISA. The concentrations of inflammatory cytokines (TNF-*α*, IL-1*β*, and IL-6) were measured by ELISA kits (Abcam, Cambridge, USA) according to the manufacturer's instruction.

### 2.12. Statistical Analysis

Data were presented as mean ± standard deviation from 3 experiments. SPSS 16.0 software was used to assess the statistical significance. The difference between different groups was tested by Student's *t* test. *p* < 0.05 indicated statistically significance.

## 3. Results

### 3.1. NGF Induces Neurite Outgrowth and TH mRNA expression in PC12 Cells

**Figure 1:**
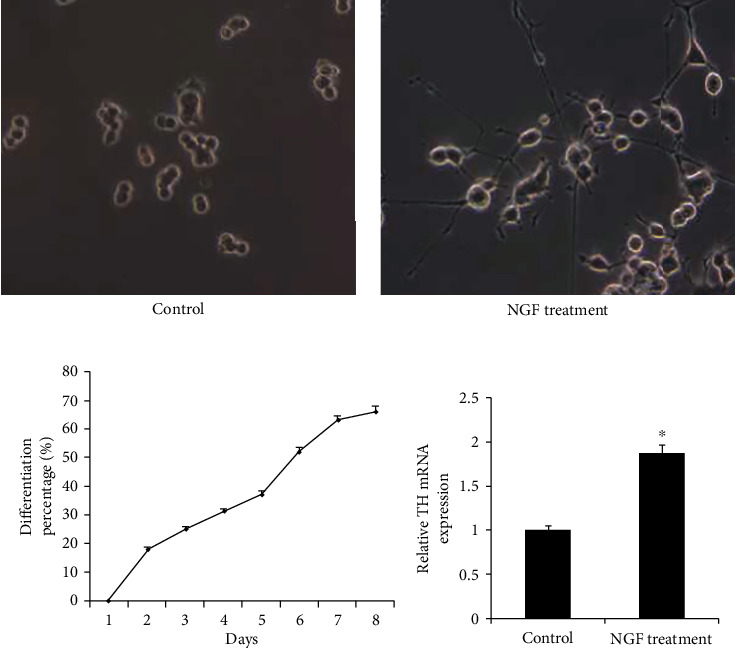
NGF induced PC12 cell differentiation. (a) Representative image of PC12 cells exhibited typical round morphology before NGF treatment (left). Differentiated PC12 cells showed the extensive neurite network after NGF treatment (right) (b) The percentage of cells with neurites. (c) The TH mRNA expression was upregulated in NGF-treated PC12 cells compared with the untreated cells using the RT-qPCR method. ^∗^*p* < 0.05. Error bars indicate standard deviation (SD).

PC12 cells were typical round morphology cells and had no visible neurites before NGF treatment. After exposure to NGF for 7 days, an extensive neurite network was formed (Figure 1(a)). In order to quantify differentiation, the percentage of neurite-bearing cells were counted after 7 days of differentiation. As shown in Figure 1(b)be, the percentage of the neurite-bearing cells increased after NGF treatment and reached about 70% after 7 days. TH enzyme plays an important role in PD because of its activity in dopamine biosynthesis, which was widely used as a marker of dopaminergic neurons. RT-qPCR showed that TH mRNA level was increased in differentiated compared with control PC12 cells (Figure 1(c)), suggesting NGF induced PC12 cell differentiation.

### 3.2. MPP+ Induces Apoptosis and ROS Production in Differentiated PC12 Cells

**Figure 2:**
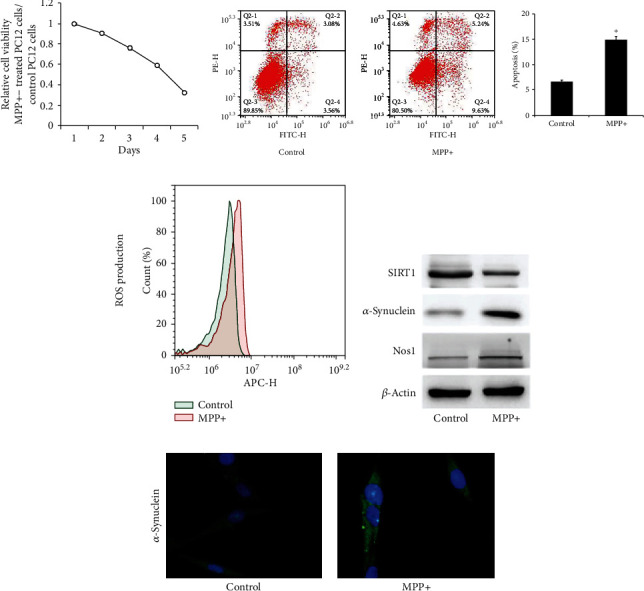
MPP+-induced neurotoxicity in the differentiated PC12 cell model. (a) Differentiated PC12 cells were treated with MPP+ (800 *μ*M, 24 h), and MTT assays were used to determine cell viability. The results showed that cell viability decreased significantly after MPP+ treatment for 4 days. (b) Cells were stained with annexinV/PI, and apoptosis percentage was analyzed by flow cytometry. The results showed that the apoptosis percentage was increased significantly after MPP+ treatment. The upper and lower right quadrants (late and early apoptosis) were used for estimation of the apoptotic percentage. (c) Cells were stained with the CellROX Deep Red Reagent, and ROS generation was determined by flow cytometry. ROS production was increased after MPP+ treatment. (d) Western blot showed that MPP+ treatment decreased the SIRT1 protein expression and increased *α*-synuclein and the Nos1 expression. (e) Immunofluorescence of *α*-synuclein in control and MPP+-treated PC12 cells. ^∗^*p* < 0.05. Error bars indicate standard deviation (SD).

MPP+ is a common dopaminergic neurotoxin that is widely used in in vitro PD models. According to previous report [16], differentiated PC12 cells were treated with 800 *μ*M MPP+ for 24 hours. The MTT assay had been preciously used to examine the cell viability of MPP+-treated PC12 cells [16–18]. The MTT assay showed that cell viability was decreased after MPP+ treatment (Figure 2(a)). Apoptosis (annexin V/PI staining) analysis showed that MPP+-treated PC12 cells had higher apoptosis percentage compared to the control cells (Figure 2(b)). We next examined the effects of MPP+ on ROS production using CellROX Deep Red staining. Flow cytometry results showed that MPP+ increased the fluorescence intensity of CellROX staining, indicating MPP+ increased ROS production in PC12 cells (Figure 2(c)).

### 3.3. MPP upregulates miR-141-3p and downregulates SIRT1 in Differentiated PC12 Cells

**Figure 3:**
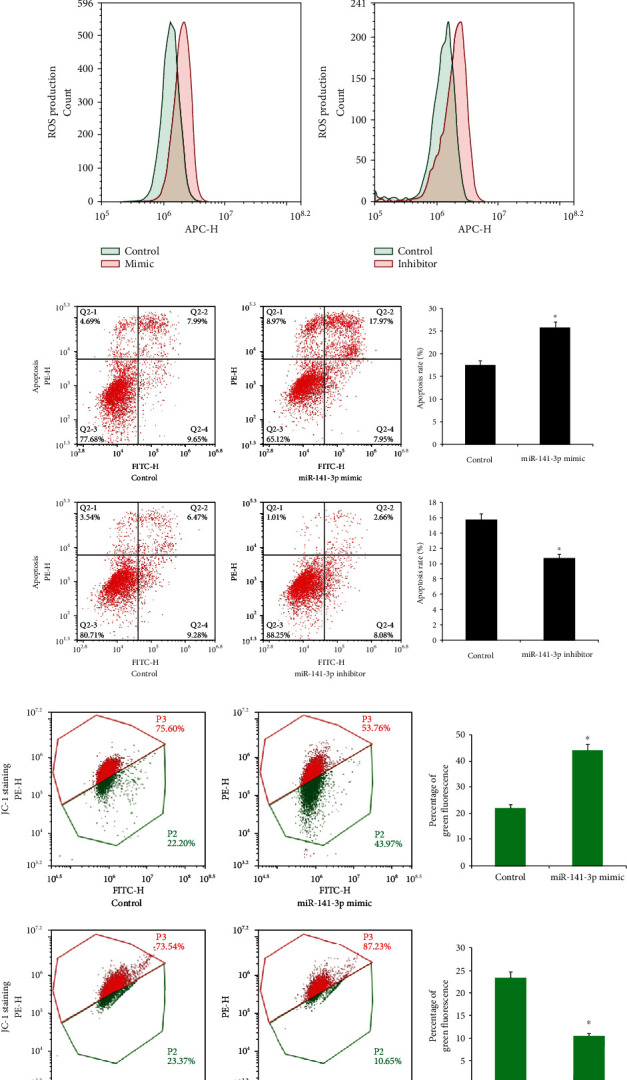
miR-141-3p regulated apoptosis, ROS production, and mitochondrial membrane potential in the PD model. (a) Differentiated PC12 cells were treated with MPP+ (800 *μ*M, 24 h). RT-qPCR showed that MPP+ treatment increased the miR-141-3p expression significantly. (b) RT-qPCR confirmed the transfection efficiency of the miR-141-3p mimic and inhibitor. (c) RT-qPCR results showed that the miR-141-3p expression increased significantly after miR-141-3p mimic transfection compared with the control group, while the miR-141-3p expression decreased significantly after the miR-141-3p inhibitor transfection. (d) CellROX Deep Red staining showed that miR-141-3p mimic transfection increased ROS production, while miR-141-3p inhibitor transfection decreased ROS production. (e) Annexin V/PI staining showed that miR-141-3p mimic increased the apoptosis percentage while the miR-141-3p inhibitor decreased the apoptosis percentage. ^∗^*p* < 0.05. Error bars indicate standard deviation (SD).

After exposure to MPP+ for 24 hours, expression of Nos1 and *α*-synuclein, two biomarkers of PD progression, were significantly increased, and the SIRT1 protein was decreased in PC12 cells (Figure 2(d)). Upregulation of *α*-synuclein was also confirmed by immunofluorescence (Figure 2(e)). RT-qPCR results showed that MPP+ also significantly increased the miR-141-3p level (Figure 3(a)). To explore the involvement of miR-141-3p in the PC12 PD model, miR-141-3p mimic and miR-141-3p inhibitor were transfected into PC12 cells, and RT-qPCR was used to estimate the transfection efficiency. The miR-141-3p expression was increased significantly after miR-141-3p mimic transfection and decreased after miR-141-3p inhibitor transfection (Figure 3(b)). Then, we checked changes of ROS production, apoptosis percentage, and *Δψ*m using flow cytometry. Mitochondrial dysfunction is a significant character of PD, and JC-1 staining was used to monitor the change of mitochondrial membrane potential (*Δψ*m). JC-1 emits red fluorescence when *Δψ*m is maintained at a normal level and turns into green fluorescence when *Δψ*m is decreased. Our results showed that transfection of miR-141-3p mimic increased the ROS production and apoptosis, and downregulated *Δψ*m. The miR-141-3p inhibitor decreased ROS production, apoptosis, and upregulated *Δψ*m (Figures 3(c)–3(e)).

### 3.4. SIRT1 Is a Direct Target of miR-141-3p in Differentiated PC12 Cells

**Figure 4:**
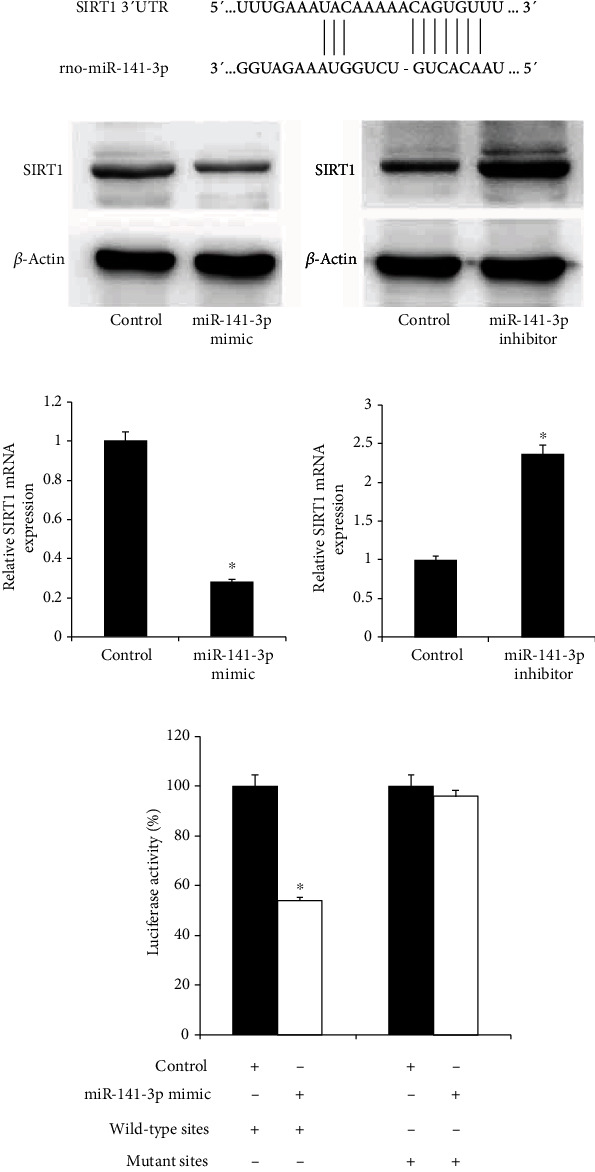
miR-141-3p negatively regulated SIRT1 by direct binding to its 3′-UTR. (a) The predicted binding sites of miR-141-3p and SIRT1. (b) The miR-141-3p mimic and inhibitor was transfected into PC12 cells. Western blot showed that miR-141-3p mimic decreased the SIRT1 protein. The miR-141-3p inhibitor increased the SIRT1 protein. (c) RT-qPCR showed that miR-141-3p mimic decreased SIRT1 mRNA. The miR-141-3p inhibitor increased SIRT1 mRNA. (d) SIRT1 3′-UTR-wild-type or SIRT1 3′-UTR-mutant reporter was transfected along with miR-141-3p mimic into PC12 cells. The luciferase activity was decreased significantly when miR-141-3p mimic was cotransfected with SIRT1 3′-UTR-wild-type reporter. ^∗^*p* < 0.05. Error bars indicate standard deviation (SD).

We used TargetScan 7.1 to predict the binding sites of miR-141-3p on the SIRT1 3′-UTR region. The results showed that there was a putative binding site on SIRT1 3′-UTR (Figure 4(a)). To confirm the potential regulatory effect of miR-141-3p on SIRT1, miR-141-3p mimic and miR-141-3p inhibitors were transfected into differentiated PC12 cells. miR-141-3p mimic downregulated the SIRT1 expression at both mRNA and protein levels. miR-141-3p inhibitor upregulated the SIRT1 mRNA and protein expression (Figures 4(b) and 4(c)). To further validate their relationship, the luciferase reporter assay was performed. Transfection of the miR-141-3p mimic and SIRT1 wild-type or mutant reporter plasmids showed that miR-141-3p downregulated the luciferase activity of SIRT1-wild-type reporter compared with the control group. There was no significant change of SIRT1-mutant reporter (Figure 4(d)).

### 3.5. SIRT1 Attenuates MPP+-Induced Neurotoxicity in Differentiated PC12 Cells

**Figure 5:**
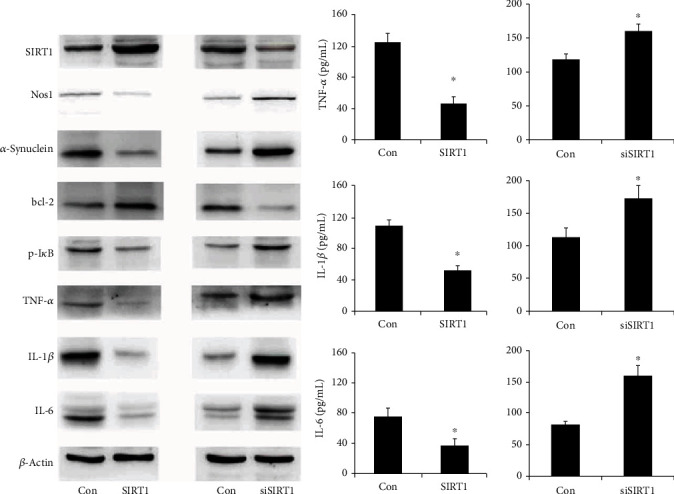
SIRT1 regulated the related protein expression. (a) Western blot results showed that the SIRT1 overexpression downregulated Nos1, p-I*κ*b, TNF-*α*, IL-1*β*, and IL-6 and upregulated the Bcl-2 protein expression. SIRT1 siRNA upregulated Nos1, p-I*κ*B, TNF-*α*, IL-1*β*, and IL-6 and downregulated Bcl-2 levels in MPP+-treated PC12 cells. (b) The effect of SIRT1 on the secretion of inflammatory factors was determined by ELISA. TNF-*α*, IL-1*β*, and IL-6 levels decreased after the SIRT1 overexpression. SIRT1 depletion upregulated TNF-*α*, IL-1*β*, and IL-6 levels. ^∗^*p* < 0.05. Error bars indicate standard deviation (SD).

**Figure 6:**
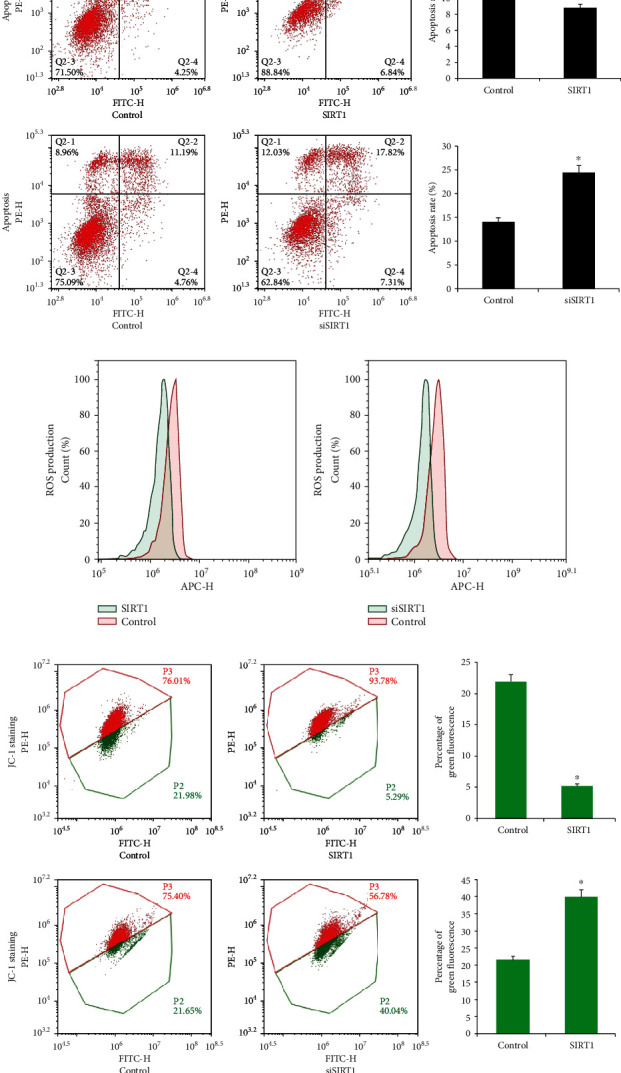
SIRT1 attenuates MPP+-induced neurotoxicity, ROS production, and loss of *Δψ*m. SIRT1 plasmid and SIRT1 siRNA were transfected into PC12 cells, respectively. Apoptosis percentage, ROS production, and *Δψ*m were analyzed through flow cytometry. (a) The annexin V/PI staining assay showed that the SIRT1 overexpression significantly decreased the apoptosis percentage, while SIRT1 depletion increased the apoptosis percentage in MPP+-intoxicated PC12 cells. (b) CellROX Deep Red staining showed that the SIRT1 overexpression inhibited ROS production, while SIRT1 depletion increased ROS production in MPP+-treated PC12 cells. (c). The JC-1 staining assay showed that SIRT1 transfection increased *Δψ*m, while SIRT1 siRNA knockdown decreased *Δψ*m in MPP+-treated PC12 cells. ^∗^*p* < 0.05. Error bars indicate standard deviation (SD).

SIRT1 plasmid and siRNA were transfected into PC12 cells, and transfection efficiency was confirmed using western blot. As shown in Figure 5(a), the SIRT1 protein expression was increased after transfection and decreased after SIRT1 knockdown. We firstly examined the effect of SIRT1 on apoptosis. Annexin V/PI staining showed that the SIRT1 overexpression reduced the apoptosis percentage while SIRT1 siRNA increased the apoptosis percentage (Figure 6(a)), suggesting SIRT1 suppressed MPP + -induced apoptosis in differentiated PC12 cells. The results also showed that the SIRT1 overexpression reduced the CellRox fluorescence intensity and SIRT1 depletion enhanced the CellRox fluorescence intensity in differentiated PC12 cells (Figure 6(b)), indicating that SIRT1 inhibited ROS formation in the PC12 PD model. As shown in Figure 6(c), SIRT1 overexpression upregulated *Δψ*m and SIRT1 siRNA downregulated *Δψ*m. The above data suggested SIRT1 relieved MPP+-induced neurotoxicity in differentiated PC12 cells.

### 3.6. SIRT1 Regulates Nos1, Bcl-2, p-I*κ*b, *α*-Synuclein, and Inflammatory Cytokines in Differentiated PC12 Cells

To investigate the potential mechanism of SIRT1 in the in vitro PC12 PD model, several related proteins were screened. Western blot results showed that the SIRT1 overexpression decreased Nos1, p-I*κ*b, TNF-*α*, IL-1*β*, and IL-6, while increased Bcl-2 levels. Conversely, SIRT1 depletion upregulated Nos1, p-I*κ*b, TNF-*α*, IL-1*β*, IL-6, and downregulated Bcl-2 levels (Figure 5(a)). Blot densitometry was confirmed using ImageJ (Supplementary Figure 1A). TNF-*α*, IL-1*β*, and IL-6 are secreted inflammatory cytokines, which were also examined by ELISA. As shown in Figure 5(b), the expression of TNF-*α*, IL-1*β*, and IL-6 decreased in SIRT1 overexpression cells. SIRT1 depletion showed the opposite effect on the secretion of TNF-*α*, IL-1*β*, and IL-6.

### 3.7. miR-141-3p Regulated *Δψ*m by Targeting SIRT1

**Figure 7:**
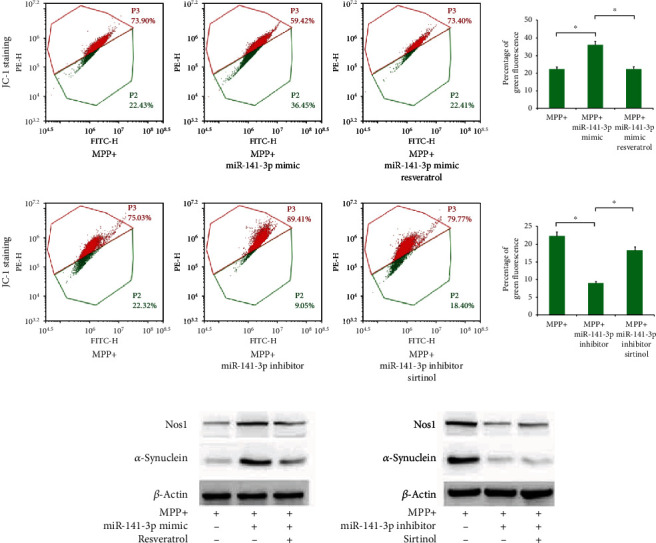
miR-141-3p regulated Nos1 and *Δψ*m through SIRT1 in PC12 cells. (a) Transfection of miR-141-3p mimic decreased *Δψ*m, and SIRT1 activator resveratrol increased *Δψ*m in MPP+-treated PC12 cells. The miR-141-3p inhibitor increased *Δψ*m, and the SIRT1 inhibitor sirtinol decreased *Δψ*m. (b) Western blot showed that miR-141-3p mimic upregulated the Nos1 level, which was downregulated by resveratrol treatment. The miR-141-3p inhibitor downregulated the Nos1 protein level, which was slightly upregulated by sirtinol treatment. ^∗^*p* < 0.05. Error bars indicate standard deviation (SD).

To further confirm whether miR-141-3p-regulated biological function by targeting SIRT1, SIRT1 activator (resveratrol) and inhibitor (sirtinol) were used in PC12 cells together with the miR-141-3p mimic and inhibitor, respectively. JC-1 staining showed that miR-141-3p mimic downregulated *Δψ*m, and that resveratrol treatment restored the *Δψ*m in PC12 cells. Conversely, the miR-141-3p inhibitor maintained *Δψ*m at a high level, and sirtinol downregulated the *Δψ*m (Figure 7(a)). These results indicated miR-141-3p regulated *Δψ*m in PC12 cells at least in part by targeting SIRT1. In addition, we examined change of Nos1. Western blot showed that miR-141-3p mimic upregulated the Nos1 protein level, which was downregulated by resveratrol treatment. In contrast, the miR-141-3p inhibitor downregulated the Nos1 protein expression, which was partially restored by sirtinol treatment (Figure 7(b)). Blot densitometry was confirmed using ImageJ (Supplementary Figure 1B).

## 4. Discussion

The etiology and pathogenesis of PD have not been fully elucidated but it is believed that both genetic and environmental factors are involved. Apoptosis, inflammation, mitochondrial dysfunction, and ROS production have been reported to be associated with PD-related neuron degeneration. In present study, we used MPP + -treated PC12 cells as an in vitro PD model to investigate the roles and mechanisms of the miR-141-3p/SIRT1 axis in PD pathogenesis. MPP+ is a parkinson inducing agent, and MPP+-intoxicated PC12 cells have been widely used for exploration of dopamine degeneration in PD. Our results showed that MPP+ treatment suppressed cell viability, induced cell apoptosis, and increased ROS production in differentiated PC12 cells, which mimicked the characteristics of neurons during PD progression.

Recently, miRNAs have been implicated in PD pathogenesis. For example, miR-410 has shown neuroprotective effects in a 6-OHDA cellular model of PD, including increased neuronal cell viability and decreased ROS production, apoptosis, and caspase-3 activity [14]. miR-190 has been found to alleviate neuronal damage and inhibit inflammation by regulating Nlrp3 in the MPTP-induced PD mouse model [19]. MPP+-induced miR-141-3p upregulation has been implicated in a recent study [15], indicating that miR-141-3p might involve in the progression of PD. miR-141-3p has been reported to inhibit or promote proliferation and metastasis in human cancers, which depends on the type of cancer. miR-141-3p upregulation was found to repress T-ALL cell proliferation and promote cell apoptosis [20]. miR-141-3p functions as a tumor suppressor by targeting ZFR in non-small-cell lung cancer [21]. miR-141-3p inhibits cell proliferation, migration, and invasion by targeting TRAF5 in colorectal cancer [22]. The miR-141-3p/KLF9 axis promotes the growth of prostate cancer [23]. miR-141-3p has also been found to promoted bladder cancer proliferation [24]. However, the roles and mechanisms of miR-141-3p in PD pathogenesis remain unclear. In this study, we found that MPP+ significantly upregulated miR-141-3p in differentiated PC12 cells. miR-141-3p mimic increased neurotoxicity, increased ROS production, and decreased *Δψ*m, while the miR-141-3p inhibitor protected PC12 cells from MPP+-induced toxicity. These results indicated that miR-141-3p might promote PD development.

We also verified the direct regulation of miR-141-3p on SIRT1. miR-141-3p mimic reduced and the miR-141-3p inhibitor increased the SIRT1 expression. Their relationship was further supported by the luciferase reporter assay. SIRT1 is an NAD+-dependent mitochondrial deacetylase that is widely expressed in human tissues including the adult brain. SIRT1 has reported neuroprotective activity in various neurodegenerative diseases, including Huntington's disease (HD), Alzheimer's disease (AD), and PD [25]. SIRT1 has also been found to regulate key cellular processes including antioxidant defense [13]. The SIRT1 overexpression was reported to improve cell survival in the in vitro Huntington's disease model [26] and SIRT1 activation played a neuroprotective role in the 6-OHDA-induced AD model [27]. Several reports have indicated the regulatory effect of miR-141 on SIRT1. The miR-141 overexpression induced the p53 protein expression through directly targeting SIRT1 and then accelerate neural apoptosis [28]. In the HepG2 cell model of hepatic steatosis, the miR-141 overexpression reduced cell survival through targeting the SIRT1/AMPK pathway [29]. In hepatocytes, miR-141 suppressed autophagic response and hepatitis B virus replication via targeting SIRT1 [30]. However, the regulatory effect of miR-141 on SIRT1 in PD has not been reported. In the present study, we found that MPP+ treatment reduced the SIRT1 expression, which was in accord with the accompanying increasein miR-141-3p expression. In addition, the SIRT1 overexpression attenuated the neuronal injury in MPP+-intoxicated PC12 cells. SIRT1 downregulated the Nos1 protein expression, which may create nitric oxide that contributes to neurodegeneration in PD. These results suggest the neuroprotective role of SIRT1 in the PC12 PD model, which are consistent with previous reports.

Mitochondrial dysfunction and oxidative stress are closely associated with apoptosis and PD-related neurodegeneration [31, 32]. MPP+ inhibits mitochondrial complex I, reducing mitochondrial ATP output, which leads to the formation of ROS [33, 34]. Our results showed that SIRT1 reduced ROS production and maintained *Δψ*m in the MPP+-intoxicated PC12 cell model. Neuroinflammation is a vital step in accelerating the degeneration and loss of neurons, and we found that SIRT1 suppressed the expression of inflammatory cytokines including TNF-*α*, IL-1*β*, and IL-6. In addition, SIRT1 downregulated p-I*κ*B and upregulated the Bcl-2 expression. Phosphorylation of I*κ*B indicated that SIRT1 could activate the NF-*κ*B signaling, which could induce inflammatory cytokines including IL-6, IL-1*β*, and TNF-*α*. Thus, it is possible that SIRT1 induced these cytokines through the NF-*κ*B activation. Bcl-2 could protect the mitochondrial function and inhibit neuron apoptosis [35, 36]. In addition, we found that the SIRT1 overexpression inhibited the aggregation of *α*-synuclein, and SIRT1 depletion promoted the aggregation. As reported, *α*-synuclein is in its monomeric state in normal neuronal cells. Aggregation of *α*-synuclein from its monomeric to oligomeric form leads to structural conformation changes in the protein that mediate the toxic effects of *α*-synuclein within neuronal cells [37]. Collectively, our data demonstrated that SIRT1 is a neuroprotective protein in this PD model.

SIRT1 activator (resveratrol) and SIRT1 inhibitor (sirtinol) provided additional evidence that miR-141-3p promoted neurotoxicity by targeting SIRT1 in this PD model. In cells transfected with miR-141-3p mimic, *Δψ*m was rescued after treatment with SIRT1 activator. The levels of Nos1 were also restored. Experiments with the miR-141-3p inhibitor and SIRT1 inhibitor yielded similar results. These results indicated that miR-141-3p aggravated PD-related neurotoxicity and mitochondrial dysfunction by directly targeting SIRT1.

In conclusion, the current study revealed a novel role of miR-141-3p by linking its regulation of SIRT1 with apoptosis, inflammation, and mitochondrial function. Our in vitro model provide insight into the mechanisms of miR-141-3p/SIRT1 in PD progression. Our data showed the importance of miR-141-3p/SIRT1 in modulating neuronal cell behaviors. Targeting miR-141-3p/SIRT1-mediated mitochondrial dysfunction might be an effective therapeutic approach for PD.

## Data Availability

The data that support the findings of this study are available on request from the corresponding author.

## Supplementary Materials

Supplementary Materials Supplementary Figure 1. Densitometric analysis of western blot. (a) Densitometric analysis of western blot bands in Figure 5(a). The relative expression of proteins was analyzed using ImageJ. B. Densitometric analysis of western blot bands in Figure 7(b). The relative expression of proteins was analyzed using ImageJ. ^∗^*p* < 0.05. Error bars indicate standard deviation (SD).
