# Supplementary figures and images for: Mir-141-3p Regulates Apoptosis and Mitochondrial Membrane Potential via Targeting Sirtuin1 in a 1-Methyl-4-Phenylpyridinium in vitro Model of Parkinson's Disease
Source: Biomed Res Int. 2020 Nov 6;2020:7239895. doi: 10.1155/2020/7239895 (PMC7666638; doi:10.1155/2020/7239895)

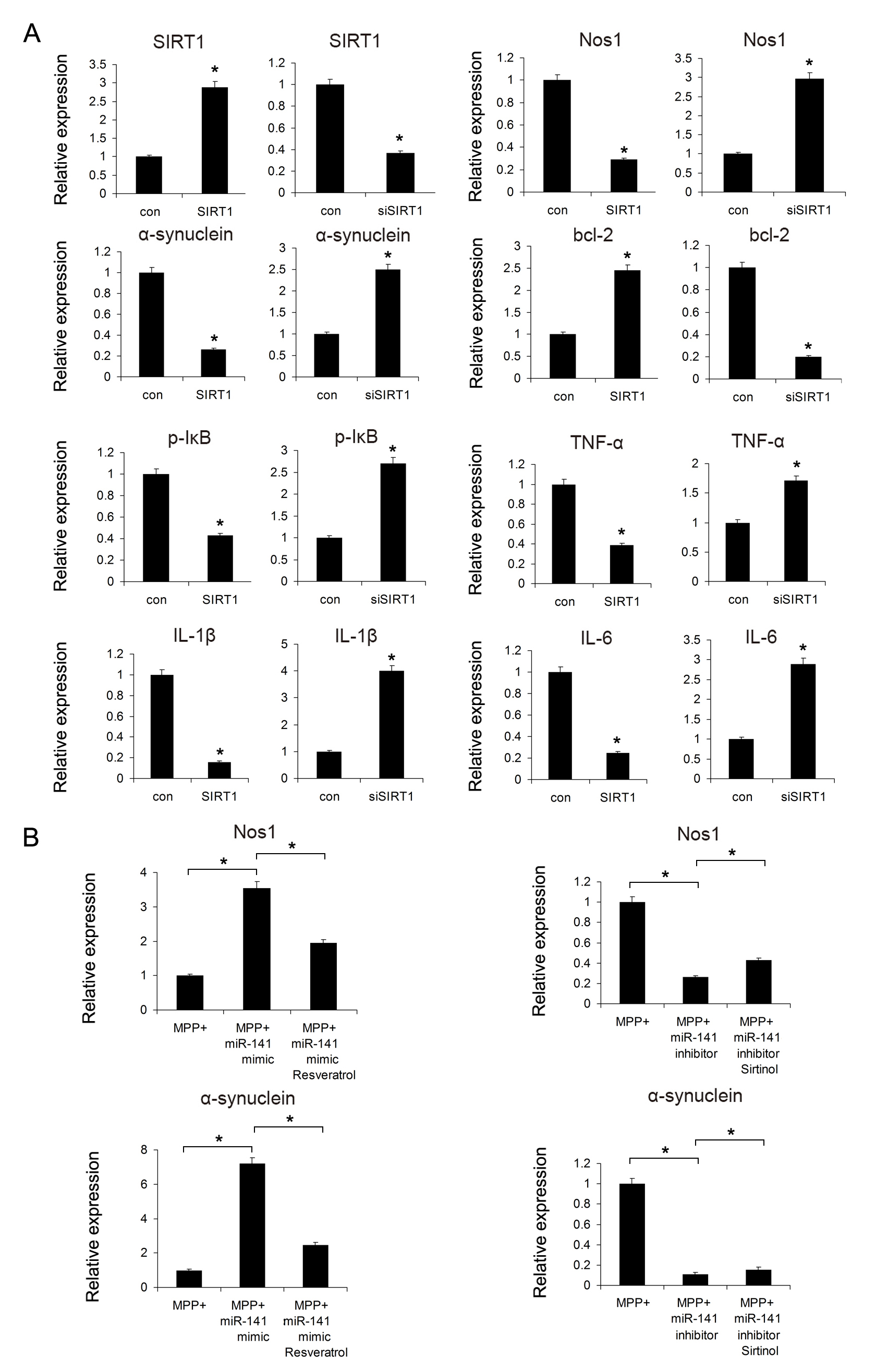

Supplement: Supplementary Materials — Supplementary Figure 1. Densitometric analysis of western blot. (a) Densitometric analysis of western blot bands in Figure 5(a). The relative expression of proteins was analyzed using ImageJ. B. Densitometric analysis of western blot bands in Figure 7(b). The relative expression of proteins was analyzed using ImageJ. ∗p < 0.05. Error bars indicate standard deviation (SD). [file 7239895.f1.zip › Supplementary Figure 1.jpg]
